# Religious Attitudes in Adolescents with Obsessive Compulsive Symptoms OCS and Disorder OCD: Religious Attitudes in Obsessive Compulsive Symptoms

**DOI:** 10.5539/gjhs.v4n6p216

**Published:** 2012-10-28

**Authors:** Ahmed Rady, Hoda Salama, Mervat Wagdy, Ahmed Ketat

**Affiliations:** 1 Department of Psychiatry, Alexandria University, Alexandria, Egypt; 2 High Institute of Public Health, Alexandria, Egypt; 3 Alexandria University Hospitals, Alexandria, Egypt

**Keywords:** obsession, compulsion, OCD, lyeton, religion, adolescent

## Abstract

**Background:**

Mental health professionals observed booming in prevalence of obsessive compulsive symptoms among children and adolescents. Our epidemiological study aims at estimating the prevalence of obsessive symptoms and obsessive compulsive disorder among secondary school students and, as a secondary research objective, to assess religious attitudes among those patients.

**Materials:**

The study is cross sectional conducted on 1299 secondary school students, adequate sample size estaimated on a prevalence of 2% for Obsessive Compulsive Disorder (OCD) in literature. Equal samples were recruited from the 3 educative zones in Alexandria Governorate. Obsessive compulsive symptoms were assessed by the Lyeton obsessive inventory child LOI-CV, the Arabic version that has been validated and tested for reliability in Egyptian culture. Those scoring 35 or above were subjected to the Mini International Neuropsychiatric Interview for children MINI-KID Arabic. Patients with OCD had their diagnosis confirmed by psychiatric interview to assure fulfillment of criteria of OCD according to DSM IV –TR criteria. A standardized self reporting questionnaire was designed to assess religious attitudes.

**Results:**

Among the studied sample (n=1299), 201 students were scored > 35 on LOI-CV i.e. 15.5% of the total sample have OCS The prevalence of OCD among studied sample was 2.2% as 29 students from the OCS students were fulfilling diagnostic criteria for OCD according to DSM-IV TR. Religious practicing attitudes were 93.1% and 79.6% in adolescents with obsessive compulsive disorder OCD and obsessive compulsive symptoms OCS respectively with no difference (X^2^=0.07)

**Conclusion:**

There’s a high prevalence rate of obsessive symptoms among adolescents, such finding highlights the necessity and need of public awareness and screening of adolescents for early detection and management. Religious attitude didn’t show significant difference among adolescents showing only obsessive compulsive disorder or those showing only obsessive compulsive symptoms

## 1. Introduction

The World Health Organization rates OCD as one of the top 20 most disabling diseases (Falment et al., 1988 and Zohar et al., 1999). Though it may persist if left untreated, yet effective and evidence based psychological as well as drug treatments are available (Thomsen et al., 1999; Skoog et al., 1999; Wittchen et al., 2005). Early epidemiological studies report prevalence rates of 0.8% in adults and 0.25% in 5-15 year old children. In the last decade, the prevalence of OCD symptoms in the general population has been found to be remarkably high (Heyman et al., 2001; Carno et al., 1988; Regier et al., 1988). Until 1984 the most quoted figure was 0.05%. However, since 1984, 3 studies carried out in North America found prevalence of OCD in the general population to be greater than 2% which is 40 times higher than the earlier estimation from 1950. A multicentre study carried out in to assess OCD prevalence to be approximately 2% in the USA, Canada, Latin America & Puerto Rico, the findings where the same in Europe and new Zealand, while in Asia it was found to be 1.9% (Kessler et al., 2005; Bijl et al., 2005; Wittchen et al., 1992; Lee, 1990).

OCD is a global problem, as the estimated total number of population who suffered from the disorder appears to be at least 50 million worlds wide (Stefanson, 1991). OCD is ranked as 2^nd^ most prevalence psychiatric disorder, although that it is still underestimated world widely, it may be the ego dystonic nature of the disorder that enforce the sufferer to disguise or be ashamed from their symptoms, and they will not reveal their obsessive and or compulsive symptoms till they are asked directly! Young people with the disorder perceive their symptoms as embarrassing and do not disclose them unless specifically asked. Therefore, OCD in this age group often remains unrecognized and untreated. The associated distress and developmental handicap are avoidable as effective treatments are available. There is evidence that early detection and intervention improve outcome (Stefanson, 1991; Jose, 2000). The aim of this work is to epidemiologically assess the prevalence of obsessive compulsive symptoms, obsessive compulsive disorder among secondary school Students and to study different presenting obsessive and compulsive symptoms.

## 2. Subjects and Methods

Cross sectional study design was adopted. The study sample was calculated using the computer package: Epi-Info, based on an OCD prevalence estimate of 2 % in literatures. The determined minimum sample size required was 1299 students. Type I statistical error alpha was set at 0.05. Out of the 7 educational zones in Alexandria three zones with the highest density of secondary school students were included in the study namely; East, Middle and West educational zones. The predetermined sample was equally allocated on the selected educational zones. Each selected zone was represented by one public school for boys and another one for girls and a mixed sex private school. One class represented each grade level in the selected schools and the equally allocated sample on each class was selected at random. All students willing to participate in the study were included. The study was conducted on the academic year from January 2009 to December 2009.

A predesigned structured self-administered questionnaire was used to collect sociodemographic data including Age, sex and grade level and socioeconomic data including family income, individual allowance, educational level of parents, occupation of parents, number of rooms and electrical appliances in home for keeping in registry.

Psychological assessment using the Arabic Version of Leyton Obsessive Inventory-Child Version (LOI-CV) It is a self-administered scale that was designed to measure obsessive compulsive symptoms and traits in children and adolescents. It is a modification of the

Original Leyton Obsession Inventory, which was modified by Berg et al. LOI-CV consists of inventory 20 questions covering: thoughts, dirt & contamination, cleanliness &tidiness, order& routine, over conscience, checking, school work, repetition, and indecision. Scoring of items ranges between 0-3 as follows:0 = the child do not have the symptoms.

1= the symptom is present with minimal interference in daily activities.
2= the symptom is present with moderate interference.
3= the symptom is present with severe interference.

The total score ranges from 0 to 60. According to authors, high scorers were identified at cutoff score 34/35. The high scorers are students with obsessive compulsive symptoms OCSs (Berg et al., 1989; Marwa, 2005; El-Rakhaway, 1992).

Psychiatric assessment using Mini International Neuropsychiatric Interview for Children (MINI-KID) was used to identify psychiatric disorders among students with OCSs. This scale was originally designed by Sheehan et al. following the same structure and format of the adult version. The MINI-KID follows the DSM-IV and ICD-10 criteria for the diagnosis of psychiatric disorders and screens for 17 Axis I disorders (mood disorders, anxiety disorders including OCD, attention deficit disorder, conduct, alcohol/substance abuse or dependence, eating disorders and psychotic disorders). The MINI-KID is a reliable and valid measure of child and adolescent psychopathology that can be administered in a short time (5-15 minutes). It use the branching logic model to reduce the number of questions asked to only those necessary to determine the presence or absence of each diagnosis It’s sensitivity and specificity is high (0.61-0.80) to very high (0.81+) for all diagnosis. The test-retest reliability of the MINI-KID is uniformly high to very high for all psychiatric disorders (Sheehan et al., 1998).

All students with LOI-CV score ≥ 35 where subjected to the Arabic Version of MINI-KID (Ibrahem, 2002). All OCD cases recruited by MINI-KID were subjected to psychiatric assessment to verify OCD diagnosis using DSM-IV-TR criteria *American Psychiatric Association: Diagnostic and Statistical Manual of Mental Disorders revised text*. Religious attitudes, whether adolescents were practicing or non practicing, was assessed by a standardized questionnaire

### 2.1 Statistical Analysis

After the data were collected, they were coded then entered into the computer. The Statistical Package for Social Sciences (SPSS-version 11.5) as well as the Epidemiological Information Package (Epi Info, 2002) were utilized for data analysis and tabulation of results. Appropriate descriptive statistics, arithmetic mean, median, and standard deviation were done. Chi square test utilized as non parametric test to compare small groups

## 3. Results

**Figure 1:**
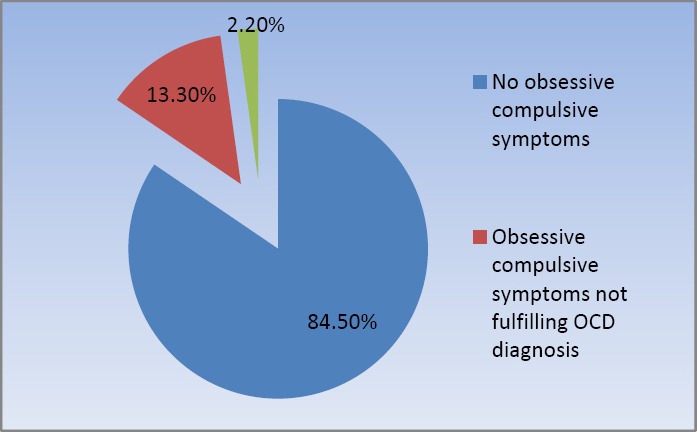
Prevalence of obsessive compulsive symptoms OCS and obsessive compulsive disorder OCD among adolescents in the studied sample

Among the studied sample (n=1299), 201 students were scored > 35 on LOI-CV i.e. 15.5% of the total sample have obsessive compulsive symptoms OCS. The prevalence of OCD among studied sample was 2.2% as 29 students from the OCS students were fulfilling diagnostic criteria for OCD according to DSM-IV TR (Figure1).

**Figure 2:**
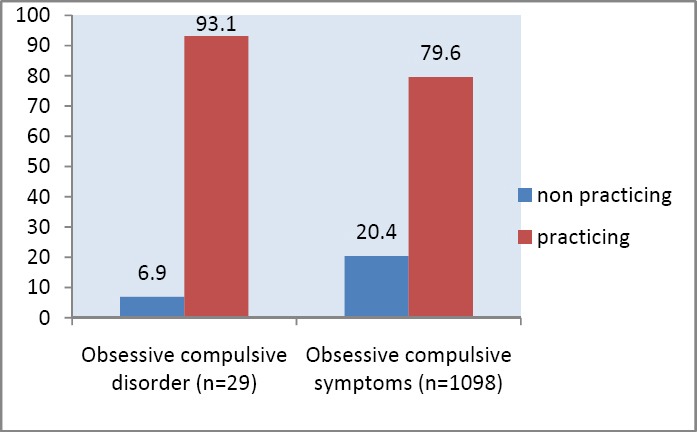
Religious attitudes (%) among adolescents with obsessive compulsive symptoms OCS and disorder OCD (No statistical difference X^2^=0.07)

Religious practicing attitude was found in 93.1% and 75.8% among adolescents with obsessive compulsive disorder OCD and obsessive compulsive symptoms OCS respectively, with no difference (X^2^=0.07) (Figure 2).

## 4. Discussion

In this study the estimated overall prevalence of OCD among secondary schools’ students was 2.2% while the prevalence for obsessive compulsive symptoms OCS was 13.3%. These results fall in the range of estimates of (0.6% to 26.3% for OCD and 14.2% to 38.7% for OCS obtained from prior studies conducted among similar target population (secondary school students) (Ismail, 1998; Hammoda, 2000). A study on the prevalence of OCS in Egyptian students in Cairo (secondary schools and university students) using General Health Questionnaire, Arabic Obsessive scale; revealed that the prevalence of OCS is 38.7% in secondary schools (Ismail, 1998). On the other hand, prevalence of OCD among the secondary school students was 26.3 % (51.3 % male and 48.7% females). These results are higher than our results. Such difference may be attributed to difference in study population, the current study was conducted on urban area rather than semiurban area (Ismail R, 1998). Another two stage epidemiological study which was conducted to investigate the point prevalence rates of OCS and OCD among secondary schools in rural areas of Shebin El-Kanater, El-Kalubia governorate in Egypt using Arabic form of Symptoms Checklist “SCL-90” to screen for OCS, in the second stage, a semi structured clinical interview based on DSM-IV diagnostic criteria was performed on the students whom considered positive in the first stage. This study was conducted among 1000 students of both genders of almost equal distribution, it was found that 9.1% were fulfilling diagnostic criteria for OCD (4.4% for male and 4.7% for females) and 18.4% were positive for OCS. The prevalence of OCD in this study was lower, this may be due to the use of different scale and to the fact that the sample used in the present study was distributed across different socioeconomic levels (Hammoda, 2000).

In a study conducted to determine the prevalence of OCS among Egyptian students among students came from the El Abasseya educational area in Cairo. The tools used in this study included the General Health Questionnaire for screening of psychiatric morbidity and the Arabic Obsessive Scale for obsessive traits. The Y-BOCS was used to determine the profile of OCS and the ICD-10 was used for diagnosis of OCD. Prevalence rate for OCS was 43.1%. Obsessive-compulsive symptoms were more prevalent among younger students, females and first-born participants. Aggressive, contamination and religious obsessions and cleaning compulsions were most common. Among participants 19% of students with OCS fulfilled *ICD-10* criteria for OCD (Okasha et al., 2000).

As regard studies conducted among similar cultures, a Saudi study of prevalence of psychological symptoms in Saudi secondary school girls, using cross-sectional study design in 10 secondary schools for girls using the Arabic version of the symptom-revised checklist 90 (SCL 90-R) for 545 female students, OCS was found to have prevalence rate of 12.3%. These results are in agreement with the result of current work despite that we conduct study among both sex and used different tool (Khaled, 2009).

It was found that in this study, adolescents with less religious tidiness are less probable to develop OCD. Most studies reported no relation between religious attitude and OCD. In an Iranian study of childhood OCD, they found no significant relation between OCD and religious practice. This finding doesn’t implicate religious practice as risk factor for OCD but it highlights the phenomenology in which OCD may presents depending on cultural and religious background in which OCD is associated with Muslim rituals of abolition and prayer (Okasha et al., 1994). Patients with OCD find it hard to terminate the abolition because of being afraid not to be clean enough to carry out prayers in lawful manner. Similarly, he or she will repeat the introductory invocations and raising arms more times than required because of the feeling not yet fully focused on God. Finally, at the end of prayer, doubts about whether he might have forgotten some words, so he start over again from beginning.

‘Wiswas’ or obsession in Arabic dialect is not conceived, in Islamic culture, as an illness that could be treated, but rather a sort of temptation by devil aiming at distracting the person from carrying out his religious duties and therefore should not be given undue attention. What may be sound is that being religiously tide is not risk for developing OCD, but being OCD with premorbid religious tidiness may indicate more suffering as symptoms usually present in this religious critical are (Okasha et al., 1994; Yossifovea, 1999; Al-Haddad, 1998; Steketee, 1991).

## 5. Conclusion

Prevalence of Obsessive Compulsive Symptoms OCS is high among adolescent age group. Early screening, detection and management may be of help for those, though undetected, still in need for further psychiatric assessment and aide. Religious tidiness and upbringing may influence phenomenology of obsessions and compulsions rather than their prevalence in the population
